# Advances in functional Neurological disorder

**DOI:** 10.1136/bmjno-2025-001285

**Published:** 2025-09-02

**Authors:** Wendy Phillips, Mark Edwards

**Affiliations:** 1 Neurology, Royal Melbourne Hospital, Melbourne, Victoria, Australia; 2 King’s College London, London, UK

**Keywords:** FUNCTIONAL NEUROLOGICAL DISORDER, CONVERSION DISORDER, TRAUMATIC BRAIN INJURY

 Functional neurological disorder (FND) is a fascinating and important area of neurological practice. It has a rich history and interfaces with philosophy, psychiatry and psychology. It is a very common condition, and one that produces very high levels of disability, impaired quality of life and health and social care costs. The disorder is still beset with myths and misunderstandings, yet the field is rapidly expanding, with real progress being made in understanding its pathophysiology, heterogeneity and effective treatment. We commissioned this topic collection ‘Advances in functional neurological disorder’ to reflect the rapid development of this important area of medicine.

The topic collection mirrors the broad scope of FND research including pragmatic clinical advice on diagnosis, patient experiences including stigma, pathophysiological advances and evidence regarding existing and novel treatments.

Regarding diagnosis, Dudley *et al* discuss the important potential mimics of functional seizures and how to spot them with confidence. This reflects an increasing maturity in the positive diagnosis of people with FND and highlights the importance of broad neurological knowledge and attention to areas where diagnostic errors can occur. Cabreira *et al* produced a diagnostic checklist to distinguish functional cognitive disorders from neurodegenerative conditions. This was developed through a literature review, Delphi study and a pilot study of consecutive patients attending a memory service in seven centres. Given the prevalence of functional cognitive disorders, such a pilot study is important to pave the way towards prospective validation studies.

Several articles deal with the very important area of patient experience of healthcare and stigma, and how these might be improved through education. Bailey *et al* report on the way in which group education sessions might improve knowledge about the illness and could reduce feelings of stigma. Joos *et al* studied the importance of locus of control and other aspects of illness perception in people with FND, something that seems to have particular importance in response to treatment and long-term prognosis. Szasz and colleagues conducted qualitative research on the lived experience of patients with FND and identified eight main themes, with the overarching ‘relationally regulated self’, that is, response to stress within a relational, regulatory context. Al-Sibahee *et al* describe attitudes and knowledge among medical students in Iraq regarding FND—in general, an essential topic in understanding how to train future healthcare professionals to deliver better care to people with FND, but also adding to the global literature on FND.

Flasbeck *et al* delve into the increasingly interesting relationship between interoception and functional neurological symptoms—this provides an important bridge between the autonomic nervous system, arousal and emotions and seems that it may have particular relevance for understanding and perhaps treating functional seizures. Kannan *et al* explore the pathophysiological similarities between FND and its common counterpart, chronic pain.

Established treatments are discussed in the analysis of the CODES trial (Goldstein *et al*), with valuable insights for future studies. We must understand how to design trials to reflect the outcomes of importance for people with FND, recognising the way in which the heterogeneity of the condition and its common comorbidities make standard randomised controlled trial design challenging to implement. Rioux *et al* describe a pilot of tailored cognitive therapy for functional cognitive disorders post-mild traumatic brain injury (as opposed to ‘standard’ post-concussion treatments), recognising the limitations of the broad ‘post-concussive’ syndromic diagnosis and the potential benefits of specifying the actual disorder present, for example, functional cognitive disorder. Saunders *et al* describe the outcomes for people with severe FND following specialist inpatient rehabilitation—essential data for understanding who should be referred to such programmes. Given the scarcity of resources in the public health system, in general, and in FND, in particular, as well as high costs incurred by the FND (largely through unnecessary investigations, as opposed to therapeutic interventions), a study (O’Mahoney *et al*) describing healthcare utilisation in people with FND will likely be very useful for those advocating for funding for clinical pathways and specific services. Finally, invaluable for those setting up services, a paper by Lehn *et al* describes treatment recommendations for healthcare professionals in Australia, including a detailed manual as supplementary material.

Finally, some exciting new approaches to treatment are described. Given the importance of the right temporo-parietal junction for agency and its involvement in FND, neuromodulation of this region in patients with FND is very compelling, and Buhler *et al* describe a pilot study demonstrating the ability to modulate the activity of this area. Central to the predictive coding model of FND is abnormal preconscious predictions about illness and a shift from automatic to goal-directed movements; virtual reality is thus a promising tool for treatment, and there is an excellent review in the collection from Brouwer *et al* outlining a research agenda.

We hope that this topic collection gives readers a sense of the dynamism and scope of current research and practice in FND. We are confident that the years to come will show the fruits of this work in better outcomes for all those affected by this serious and much neglected condition.

